# Impact of the healthcare payment system on patient access to oral anticancer drugs: an illustration from the French and United States contexts

**DOI:** 10.1186/1472-6963-14-274

**Published:** 2014-06-20

**Authors:** Laure Benjamin, Valérie Buthion, Gwenaëlle Vidal-Trécan, Pascal Briot

**Affiliations:** 1 Department of Epidemiology, Evaluation and Health Policies, University of Paris Descartes, Paris Sorbonne Cité, Paris, France; 2 School of Public Health (EHESP), Rennes, France; 3 Health Economics and Outcomes Research, GlaxoSmithKline, 100 route de Versailles, Marly le Roi, Cedex 78163, France; 4 COACTIS EA 4161, University of Lyon, 14 avenue Berthelot, Lyon 69363, Cedex 07, France; 5 Department of Public Health, Quality and Safety of care, Paris Center University Hospitals, AP-HP, 27 rue du Faubourg Saint Jacques, Paris 75014, France; 6 Department of Public Health, Faculty of Medicine, Paris Descartes University, Paris Sorbonne Cité, Paris, France; 7 Institute for Health Care Delivery Research, Intermountain Healthcare, 36 South State Street, 16th Floor, Salt Lake City, UT 84111, USA

**Keywords:** Oral, Chemotherapy, Targeted therapy, Healthcare payment system, Reimbursement, Hospital funding, Medicare Part D, Cancer

## Abstract

**Background:**

Oral anticancer drugs (OADs) allow treating a growing range of cancers. Despite their convenience, their acceptance by healthcare professionals and patients may be affected by medical, economical and organizational factors. The way the healthcare payment system (HPS) reimburses OADs or finances hospital activities may impact patients’ access to such drugs. We discuss how the HPS in France and USA may generate disincentives to the use of OADs in certain circumstances.

**Discussion:**

French public and private hospitals are financed by National Health Insurance (NHI) according to the nature and volume of medical services provided annually. Patients receiving intravenous anticancer drugs (IADs) in a hospital setting generate services, while those receiving OADs shift a part of service provision from the hospital to the community. In 2013, two million outpatient IADs sessions were performed, representing a cost of €815 million to the NHI, but positive contribution margin of €86 million to hospitals. Substitution of IADs by OADs mechanically induces a shortfall in hospital income related to hospitalizations. Such economic constraints may partially contribute to making physicians reluctant to prescribe OADs. In the US healthcare system, coverage for OADs is less favorable than coverage for injectable anticancer drugs. In 2006, a Cancer Drug Coverage Parity Act was adopted by several states in order to provide patients with better coverage for OADs. Nonetheless, the complexity of reimbursement systems and multiple reimbursement channels from private insurance represent real economic barriers which may prevent patients with low income being treated with OADs. From an organizational perspective, in both countries the use of OADs generates additional activities related to physician consultations, therapeutic education and healthcare coordination between hospitals and community settings, which are not considered in the funding of hospitals activities so far.

**Summary:**

Funding of healthcare services is a critical factor influencing in part the choice of cancer treatments and this is expected to become increasingly important as economic constraints grow. Drug reimbursement systems and hospital financing changes, coupled with other accompanying measures, should contribute to improve equal and safe patient access to appropriate anticancer drugs and improve the management and care pathway of cancer patients.

## Background

Most anticancer drugs are usually administered by intravenous (IV) route during hospitalizations including inpatient (overnight hospital stay) and outpatient hospitalizations (one-day hospital stay). IV treatments are mainly administered during outpatient hospitalizations while inpatient hospitalizations are usually used for the first administration if there is a need to manage potential immediate adverse effects such as allergic reactions, or for disabled patients requiring additional care. As a result, medical oncology activities have been structured for many years within hospital settings and many anticancer drugs are still only delivered during inpatient and outpatient hospitalizations. Nonetheless, since the end of 1990s, an increasing number of anticancer drugs have been developed for oral use, especially for the treatment of breast cancer, non-small lung cancer, colorectal cancer and prostate cancer. In a task force report on oral chemotherapy, a National Comprehensive Cancer Network (NCCN) working group has estimated that this trend would continue, since 25% of anticancer drugs currently under development are planned to be available as an oral formulation [1]. Others have estimated that 10% of current anticancer drugs are available in an oral form [2]. It has been postulated that oral anticancer drugs (OADs) may contribute to improving patients’ quality of life [3,4], since administration of OADs avoids the inconvenience of infusions, the risk of infections or extravasations, pain at the site of infusion, stress related to infusion, and visits to hospital. This route of administration allows patients to take their medication at home. The follow-up of patients receiving OADs may be still performed in hospital setting. The increasing incidence of cancer, development of more therapeutic alternatives, increasing patient involvement in disease management and treatment decisions, claimed improvements of quality of life, limitations on hospital resources, and healthcare policies have all contributed to the move towards OADs. It has been demonstrated that, assuming equivalent efficacy, patients prefer OADs to IV medication [5,6]. However, the use of OADs is still controversial in the medical community [7,8]. The main criticisms towards OADs relate to potential difficulties in the management of drug therapy, such as drug interactions, controlling treatment adherence, and managing adverse effects [1-3,7-10]. Economic considerations relating to the use of OADs may also have an impact on their prescription and usage and thus may influence patient access to these drugs [11,12]. So far, most published studies on OADs have focused on treatment adherence and safety [2-4,7,8]. Few studies have specifically investigated the economic implications related to the use of OADs [7,13,14]. Nonetheless, it seems critical to develop a global approach to this issue, since medical, organizational, financial and regulatory issues are closely interdependent. With respect to publicly available information and data, France and the United States of America (U.S.) were the two countries for which relevant published information on the oral and IV drug payment system was available. These two healthcare systems provided an opportunity to illustrate the issue of patient access to OADs which can be explained by different mechanisms. In France the economic implications of OADs were raised during roundtable discussions in 2008 [15]. In the U.S., the adoption in 2006 of the oral and IV chemotherapy parity legislation provides an opportunity to illustrate economic issues around the patient access to these drugs. The aim of this article is to discuss how the healthcare payment system (HPS) may create disincentives to the use of OADs within the French and U.S. healthcare systems. These two different healthcare systems provide an interesting basis to illustrate the common issues related to the patient access to OADS.

## Discussion

### The disincentive effect of the hospital per-case payment system: illustration from the French situation

In France, the funding of all public or private hospitals is assured by the National Health Insurance (NHI) fund according to the nature and volume of medical procedures performed. A cost-per-case mix (i.e. per-case payment system) is applied based on the type of medical activity documented in the French national hospital database (PMSI, *Programme Médicalisé des Systèmes d’Information*). Because it is compulsory to report all hospitalizations, the PMSI database, which is the basis of all hospital funding, is exhaustive. Therefore the analysis of this national database provides an opportunity to assess the contribution of chemotherapy sessions to the whole hospital activity. Chemotherapy administration is mainly performed during outpatient hospitalizations (93% versus 7% during inpatient hospitalizations). Outpatient chemotherapy sessions accounted for 7% of all hospitalizations performed in 2013 across all public and private institutions. This medical activity increased by 32% over the period 2006 to 2013 [16]. In 2013, outpatient chemotherapy sessions represented the second most frequent reason for hospital visits after hemodialysis. Outpatient chemotherapy sessions, coded according to the Diagnosis Related Group (DRG) classification as 28Z07Z, accounted for 2 221 864 outpatient hospitalizations, of which 1 511 364 (68%) were performed in public hospitals and 710 500 (32%) in private hospitals. From the published official tariffs (i.e. reimbursement amount) associated with the DRG 28Z07Z (€396.37 and €304.72 per session in public and private hospitals, respectively) [17], we estimated that the financial resources allocated by the NHI to outpatient chemotherapy sessions reached over €815 million in 2013 (Table 1). This estimation is based only on the administration of chemotherapy (outpatient hospitalizations for chemotherapy administration) and excludes the additional cost of expensive drugs that are funded by the NHI in addition to the cost-per-case-mix. This estimation excludes also the direct non-medical costs of medical transportation between hospital and patient homes and the cost of sick leave. The contribution margin (i.e. proportion of sales revenue that is not covered by variable costs and which contributes to the coverage of fixed costs) for hospitals attributable to outpatient chemotherapy sessions were estimated by comparing what NHI paid to the cost incurred by public and private hospitals. Since NHI pays more than it costs to public and private hospitals, positive contribution margin for hospitals attributable to outpatient chemotherapy sessions were estimated to be €86 million in 2013 (Table 2).

**Table 1:** Estimation of the costs induced by outpatient chemotherapy sessions in France

	**National Health Insurance perspective**		
**Sector**	**Number of outpatient chemotherapy sessions performed in 2013**	**Official unit tariff applied in 2014**^**#**^	**Cost for the National Health Insurance**
**Public**^**†**^	1 511 364	396,37 €	599 059 349 €
**Private**^**‡**^	710 500	304,72 €	216 503 560 €
**Total**	**2 221 864**	-	815 562 909 €*

^†^Public sector includes all public hospitals (university hospital centers, regional hospital centers, hospital centers and local hospitals).

^‡^Private sector includes clinics and private institutions involved in public service.

^#^Official unit tariff corresponds to official reimbursement rate applied by the National Health Insurance [17]. The basis for calculation of costs differs between the public sector and the private sector. In the private sector, doctors are self-employed and their fees are paid over and above the per-case mix, while in the public sector, physicians are employees of their institution and their fees are included in the per-case mix.

*Estimations were made from the analysis of the number of outpatient chemotherapy sessions performed in the public and private sectors in France in 2013. The cost supported by the National Health Insurance (NHI) was estimated by multiplying the number of sessions performed in each sector by the corresponding official tariff applied by the NHI. These reimbursement rates are published annually in the Official Journal of the French Republic.

**Table 2:** Estimation of the contribution margin accrued by the outpatient chemotherapy sessions in France

	**Hospital perspective**								**National Health Insurance perspective**	**Hospital perspective**
	**Total hospital costs associated with outpatient chemotherapy sessions (DRG 28Z07Z)**			Costs associated with expensive drugs**		Costs associated with physician fees**		Costs attributable to the administration of chemotherapy***	**Amount/fee paid by the National Health Insurance**	**Net contribution margin for public and private hospitals**^**+**^
	**Number of sessions performed in 2013#**	**Unit hospital cost per DRG**^**†**^	Total cost*	**Cost associated with expensive drugs**	**Total cost**	**Cost associated with physician fees**	**Total cost associated with physician fees**			
**Sector**	**(A)**	**(B)**	**(A x B)**	**(C)**	**(A x C)**	**(D)**	**(A x D)**	**(A x B) - ((A x C)+ (A x D)) = (E)**	**(F)**^**$**^	**(F) – (E)**
**Public**	1 511 364	922€	1 393 477 068 €	546 €	825 355 880 €	NA	NA	568 121 728 €	599 059 349 €	30 937 621€
**Private**	710 500	823€	584 741 500 €	566 €	401 787 750 €	31 €	22 025 500 €	160 928 250 €	216 503 560 €	55 575 310 €
**Total**	**2 221 864**	**-**	**1 978 219 108€**	**-**	**1 227 143 630 €**	**-**	**22 025 500 €**	**729 049 978 €**	**815,562,909 €**	**86 512 931 €**

NA: Not Applicable.

DRG: Diagnosis Related Group.

^#^The number of outpatient chemotherapy sessions was obtained from the 2013 National Hospital database (PMSI, 2013) [16].

^†^The unit hospital cost per DRG represents the average cost of hospital stay for the administration of anticancer treatments during outpatient hospitalization (without overnight hospital stay). Data was obtained from the National Scale of Costs (Etude Nationale des Coûts Complets, ENCC) (ENCC 2012 version v11f [18]) which is a survey conducted in France every two years in order to document cost incurred by hospitals for each Diagnosis Related Group (DRG). For each DRG, a mean hospital cost is calculated from a sample of public and private hospitals which participate to the survey.

^*^The total cost incurred by hospitals for outpatient chemotherapy sessions (DRG 28Z07Z) was calculated by multiplying the unit hospital cost per DRG (A) by the number of sessions performed in each hospital sector (B).

^**^The unit hospital cost per DRG (^**†**^) includes the cost associated with expensive drugs for public and private sectors and the costs associated with physician fees for public sector only.

^***^To estimate the cost only attributable to the administration of a chemotherapy session, the cost attributed to expensive drugs and to physicians fees should be removed. It represents the cost incurred by hospitals for the administration of cancer treatments (excluding the cost for expensive drugs and physicians fees which are paid by National Health Insurance above the DRG).

^+^It represents the revenue received by hospitals under the activity of outpatient chemotherapy administration (expensive drugs and medical fees excluded).

^$^See Table 1.

**Figure 1:**
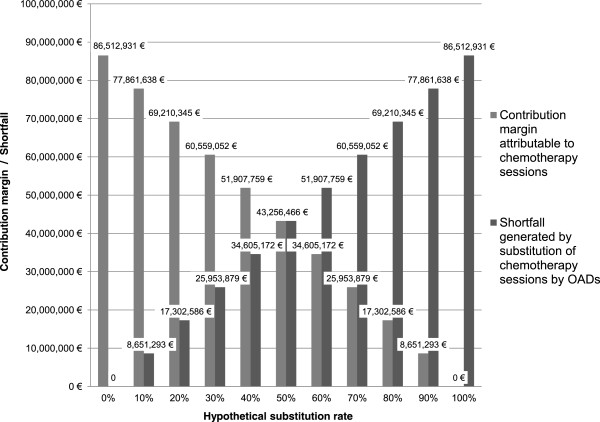
Simulation of the financial shortfall for hospitals due to the substitution of intravenous cancer treatments by oral anticancer drugs.

Based on this estimation, it is possible to evaluate the shortfall for hospitals that may arise due to the substitution of IV chemotherapy session by the use of OADs (Figure 1). The self administration of OADs by the patient instead of the use of IV anticancer drugs may generate a partial outsourcing of the management of patients to the community setting outside the hospital sector, depriving hospitals of financial revenues. The estimation presented in Figure 1 suggests that the current per-case payment system creates an incentive to perform hospital stays and therefore for the use of IV anticancer drugs rather than OADs. Indeed, prescribing OADs changes the economic paradigm of cancer treatment by partially shifting medical service from the hospital to the community setting [19]. This creates a loss of potential resources for hospitals and may create disincentives for the prescription of OADs by healthcare professionals sensitive to financial incentives [11]. The income generated by outpatient chemotherapy sessions representing an important source of revenue for hospitals, especially as they are facing budgetary deficits and constraints in an economic environment that is becoming increasingly hostile [20]. Nonetheless, some limitations may exist with this study. First the linear trend of the estimation does not reflect the natural trend that would be observed under real conditions. Second OADs are often combined with intravenous treatments and there are few situations in which oral and intravenous anticancer drugs are directly substitutable in current practice. Therefore this demonstration is only intended to illustrate the mechanisms of shortfall that may be induced by the use of OADs and does not reflect the real economic impact of the use of OADs incurred by hospitals.

### The impact of the gap in health insurance coverage on the patient access to oral anticancer drugs: illustration from the Medicare health insurance

**Figure 2:**
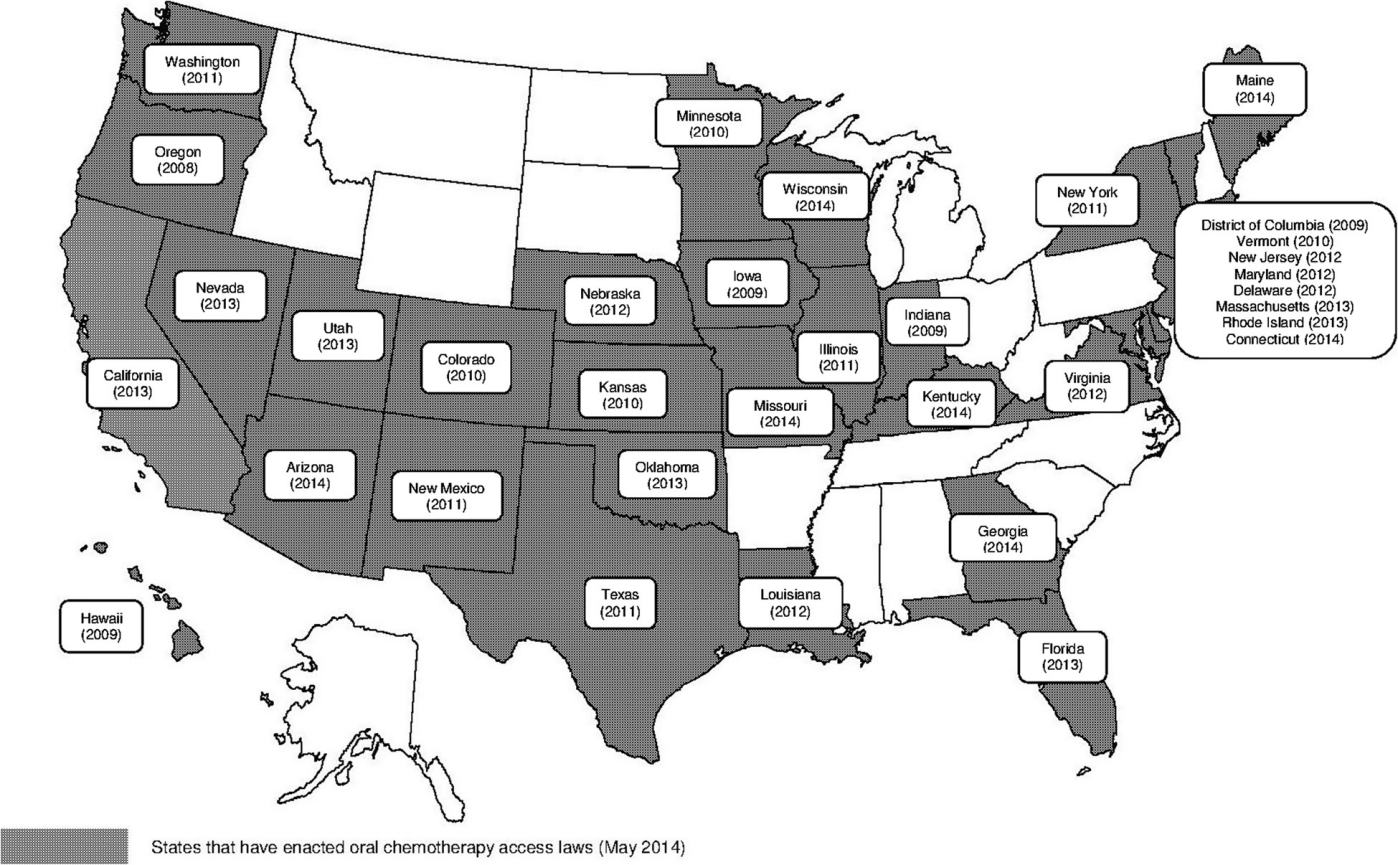
**States that have adopted the Cancer Drug Coverage Parity Act on May 2014.** Note: Map was built by the authors based on a blank map available on http://www.geo-phile.net/IMG/doc/ETATS-UNIS.doc. Legend: Oregon was the first state to require that health insurance carriers offer coverage for oral anticancer drugs equivalent to intravenous chemotherapy.

**Figure 3:**
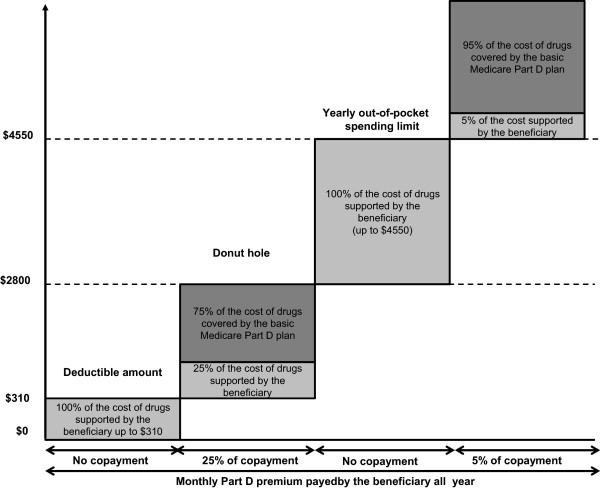
Modalities of oral anticancer drugs reimbursement by the Medicare health insurance system.

The U.S. healthcare system is based on a free-market tradition with healthcare financed by private health insurance systems for most people (63.9% in 2012) and a public health insurance system for the most vulnerable population, notably elderly, children, disabled people and women with low incomes [21]. According to the U.S Census Bureau in 2013 [21], 32.6% of the insured population was covered by public health insurance, corresponding to people aged 65 years and older or who are disabled, who are beneficiaries of the Medicare program (15.7%), and to families with low income who are eligible for the Medicaid program (16.4%). Although the Medicare program covers only a part of the U.S. insured population and therefore is not representative of all healthcare insurance systems, it is a relevant example to our study to illustrate the disparities in terms of reimbursement between oral and IV anticancer drugs. The Medicare program defines different levels of basic insurance that cover healthcare costs related to inpatient care (Part A) and to outpatient care and physician consultations (Part B). Before 2006, reimbursement for OADs was limited to oral drugs with IV equivalents covered by the Medicare standard insurance (Part B) such as capecitabine, cyclophosphamide, methotrexate, temozolomide, busulfan, étoposide, melphalan [22]. The reimbursement of OADs for limited categories of treatment has introduced disparities in patient access between oral and IV anticancer drugs [9,23]. As a consequence, OADs are covered by a prescription benefit that requires higher patient’s copayments while IV anticancer drugs are covered by a medical benefit that is more generous. As a consequence, patients need supplementary insurance which often also include, significant co-payments which may be a barrier to accessing these therapies for those with low income. In 2006, the U.S. Congress passed the Medicare Prescription Drug Improvement And Modernization Act for the beneficiaries of the Medicare program that has been adopted in 33 states in May 2014 (Figure 2) [24-26]. The Medicare Part D program was implemented in 2006 to provide Medicare beneficiaries with additional coverage for pharmaceutical care including OADs. Part D is provided under the basic Medicare program and beneficiaries must enroll in plans offered by private companies. The oral/IV chemotherapy parity legislation was adopted to provide parity between oral and IV anticancer drugs and to reduce out of pocket financial burden for patients. But despite the parity legislation, patients still have to pay out the cost of drugs before submitting an insurance claim, and they may be required to make co-payments depending on their private insurance plan’s benefits, the type of cancer and the drugs prescribed. Generally, beneficiaries pay out-of-pocket for monthly Part D premiums all year. The patient then pays 100% of the costs of drugs until expenditure reaches the deductible threshold of $310. After reaching this deductible threshold, beneficiaries pay 25% of the cost of drugs and the Part D plan covers the rest until the total reaches $2 800. Above this amount, a coverage gap called the “donut hole” occurs, when the patient has to cover the full cost of drugs until total expenditure reaches the yearly out-of-pocket spending limit of $4 550 (Figure 3). After this yearly spending limit, beneficiaries are responsible for a 5% copayment of the cost of drugs. In addition to the basic Medicare Part D coverage, patients may have additional or higher coverage by contracting with complementary Medicare Plan D plans but they will have to pay higher monthly premiums [1,27]. This pricing rule may be also common to private/commercial plans outside of Medicare.

Reimbursement may also be a concern for certain diseases for which no therapeutic option in IV form is available, such as myeloma, or for drugs which are only available in oral formulations. Therefore the price of OADs, which may be higher than that of IV chemotherapy, may be a barrier to patient access to these drugs [28,29]. The higher the price of the medication, the higher the co-pay will be. The price of anticancer drugs is such that the annual threshold set by Medicare can quickly be reached due to the number of treatment administrations needed, especially for cancers that require long therapeutic protocols. This cost is expected to increase over time as more targeted therapies, whose price is higher compared to cytotoxic chemotherapies, become available. It is therefore important that patients be clearly informed about their insurance coverage to help them evaluate if such treatments are compatible with their economic situation to ensure continuity of care throughout the treatment trajectory. Inadequate assessment of these conditions can lead patients to interrupt treatment or to delay refilling prescriptions, when faced with high out-of-pocket costs for these medicines. These situations increase the risk of treatment failure and may lead to increasing use of emergency services and hospitalizations for advanced cancer, which ultimately transfers cost from the patient to the hospital system.

In addition to the economic barrier related to co-payment, the reimbursement rule applied by the Medicare insurance program plays a role in regulating eligibility for reimbursement of OADs. The reimbursement of OADs by Medicare Part B is limited to the treatment of certain serious diseases or certain clinical emergencies, such as advanced kidney disease requiring transplantation. Four criteria determine whether OADs are reimbursed or not and are subject to prior validation by the Medicare insurance scheme on a case-by-case basis. The OADs must have been approved by the Food And Drug Administration (FDA), it must be bio-equivalent to a molecule administered intravenously, it should have the same therapeutic indications to those covered by the IV treatment and should be limited to a list of diseases that can be treated by a restrictive list of oral chemotherapy (busulfan, capecitabine, cyclophosphamide, etoposide, fludarabine, melphalan, methotrexate, temozolomide, topecan) [24]. Finally the prescription of OADs should be performed by a physician or other health care practitioner licensed to prescribe chemotherapy. This complex reimbursement scheme may require actions to assist the patient with financial issues in order to identify a co-payment program or a free drug program in certain cases. This should be initiated before treatment initiation in order to avoid drug cost being incurred by the patient.

Despite reforms to provide equal access to OADs, co-payments still exist, which means that patients’ economic conditions remains one of the several barriers to a wider use of OADs [1]. The gap in healthcare coverage results in a persistent out-of-pocket expense for every patient, potentially leading to decreased medication adherence that may reduce treatment effectiveness, as has been demonstrated by Fung et al. in patients with diabetes [30]. Suboptimal treatment effectiveness may lead to complications, diminished quality-of-life and general health status, and premature death. In this context, a cross-sectional cohort study using administrative claims data showed that 10% of cancer patients with Medicare and commercial insurance abandoned their treatment by OADs [31]. This study also showed that the rate of treatment discontinuation increased with the amount of co-payment. Another study showed that funding considerations influenced patients’ choice of treatment modalities [32]. The Medicare health insurance system is a good illustration of the potential impact of the economic environment on the use of OADs and indicates that the issue of OADs should not be considered only in medical terms but also with respect to economic aspects [25,32].

These issues need to be considered since higher drug costs increases co-payment to the patient. Out-of pocket expenditures are expected to rise as the cost of anticancer drugs increases and as the proportion of population who are underinsured rises. This issue is particularly important for cancers where there is no choice between IV and oral anticancer drugs. Restriction of availability of OADs due to economic constraints may not be the best way of controlling health care expenditures, since treatment non-adherence may induce unexpected additional costs related to complications and suboptimal treatment effectiveness.

### The impact of oral anticancer drugs on healthcare organization

**Figure 4:**
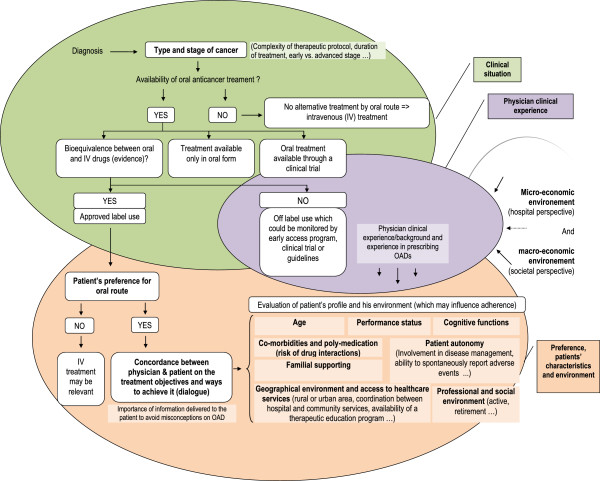
Conceptual framework of factors influencing patients’ access to oral anticancer drugs.

Apart from the economic impact associated with potential loss of revenue, the use of OADs has an impact on healthcare organization by modifying the involvement of healthcare stakeholders. Prescription of OADs generates additional activities, requires more time for medical consultation due to the need to explain the treatment protocol, as well as longer follow-up time by nurses in order to monitor treatment adherence and to manage adverse effects of treatment. These activities, which may be provided by hospital-based healthcare professionals, are not taken into account in the costing of hospital activities [15]. The additional time required during the outpatient visit when initiating OADs is necessary to ensure that the patient agrees with the treatment objective, which may help optimize patient adherence and treatment persistence. Currently, this additional time is not taken into consideration in the funding of medical activities, since physician fees are the same (€28 per specialist visit for instance in France – base price reimbursed by NHI excluding excess fees) whatever the duration of the consultation. In the same way, the time required for therapeutic education and management of adherence and adverse events is not taking into account in the payment or financing of hospital activities. These activities are often provided by nurses and are not considered as medical acts. In France, a specific funding may be affected to therapeutic education for chronic diseases (MIGAC, Missions d’Intérêt général et d’Aide à la Contractualisation) but the lack of medical staff, the disparities in practices between hospitals and the lack of recognition of therapeutic education challenge this modality of funding. In the case where these activities are not directly provided by hospitals, hospital healthcare professionals will need time to organize these activities within the community setting and to coordinate the actions of nurses in private practice, pharmacists and general practitioners to ensure optimal implementation of the chosen treatment strategy. It is therefore clear that the additional tasks associated with oral treatments can be an economic disincentive, due to the absence of compensated time for patient support in hospitals and to the lack of human resources dedicated to patient support. For these reasons, the move towards OADs challenges the current financing of the hospital healthcare system. In certain cases, the current paradigm may oppose an economic logic against a therapeutic logic for treatment decisions making. The current funding model may generate adverse effects of outsourcing of care on hospital finances, even if it is justified from a medical perspective in order to maintain or improve the patient’s quality-of-life or overall clinical state. In the French and U.S. healthcare systems, when a prescription is sent to an external party, no income is provided to the cancer centers or hospitals and the additional time dedicated to patients by healthcare professionals is not paid for [33]. Nonetheless, even if economic factors may play a role in the patient access to OADs, it is important to highlight the role of others factors. In current practice, there are clinical situations in which no alternative route of treatment administration is available depending on the type and stage of cancers or situations in which the combination of IV and oral is often required. In these situations, our hypothesis of the potential role of the economic factors on patient access to OADs is probably not valid. For situations where the choice between oral and IV route of administration is relevant, medical factors may also influence the patient access to OADs such as patients’ preference and their ability to manage their treatment (treatment adherence and cognitive functions), geographical origin of the patient and their mobility (rural or urban area), patients’ professional and social situations (active or inactive, family support or isolation), age (aspiration risks for older people when taking pills and alteration of cognitive functions, necessity of monitoring treatment adherence for pediatric cancer population), available evidence on drugs (efficacy and safety profile, bioequivalence between IV and oral forms), physicians experience in prescribing OADs (Figure 4).

## Summary

In France, all costs related to cancer care are covered in full by the National Health Insurance (NHI) in a specific program for patients with chronic disease (ALD, *Affection de Longue Durée*). In principle, there is no substantial out-of-pocket cost that may prevent patients from accessing cancer care. Nonetheless, the use of OADs deprives hospitals of sources of potential revenue because they are transferred in part to the community sectors. This transfer could influence the prescription of cancer treatment in hospitals (via indirect incentives) and thus could influence patient access to such medications. Even though OADs are covered in full by the NHI, shifts of resources from hospital to the community sector are likely to create a barrier to more widespread use of these drugs [12]. In the U.S., the patient access to OADs may be influenced in part by additional copayments and out of pocket payments incurred by the patients and the complexity of the reimbursement systems, which can discourage patients from receiving treatment with OADs. Nevertheless we should underline that the example of the Medicare program that we used in this study is not representative of all healthcare reimbursement systems in the U.S. for which there is variability especially in the private sector and for which published information remains limited. In addition, the American healthcare system is changing quickly and current reforms (e.g. Affordable Care Act, 2010) may contribute to improving patient access to healthcare in the near future. However it remains that in both systems, the absence of revenue to compensate for additional activities to manage patients treated with OADs may be a disincentive to the use of OADs.

Patient access to oral and IV anticancer drugs is an international issue whose determinants may vary from one country to another, but which raises the common issue of safe and appropriate patient access to cancer drugs [19]. As a result, the safe use of OADs seems to be determined by both the patients’ socio-economic conditions and their ability to handle insurance copayments and the healthcare system-related factors including the availability of hospital resources for providing adequate counseling, therapeutic education, and follow-up care for patients treated with OADs. Insurance payment system may have a negative impact on patient access to OADs through economic factors, in addition to patients’ care related factors such as ability to manage oral treatment, age, geographical origin, disease, stage of disease, patient’s preference.

The issue of patient access to OADs underlines the necessity to adapt healthcare services, hospital budget financing and insurance coverage to the introduction and widespread use of innovative drugs. This issue is expected to grow in importance as more and more OADs become available in the near future. Drug reimbursement systems and hospital payment are key factors contributing to increased quality and safety of cancer care by providing cancer patients with the most appropriate care. Further studies on OADs to understand the impact of non-adherence on treatment effectiveness in real life settings and to quantify healthcare resource utilization are needed. Such studies could help guide the appropriate development of new OADs. The use of OADs also highlights a major need to further develop a more complete or encompassing Health Technology Assessment (HTA) in order to anticipate the real impact of such treatments on healthcare systems. Due to the increasing number of OADs available, the economic and organizational impact of their prescription should be considered in the future in order to insure adequate access to these drugs for eligible patients when considered as a relevant treatment option for the patient. In addition, given potential interactions between factors influencing access to OADs, further research and analysis to determine the relative impact of such factors would be useful.

## Competing interests

None of the authors declare a conflicting interest. Dr Laure Benjamin is employee of GlaxoSmithKline (GSK) group of companies and holds stock or stock options or restricted shares (GSK). This work was initiated by Dr Laure Benjamin when she was a doctoral fellow, whose research was partly financed by the Association Nationale pour la Recherche et la Technologie (ANRT, Paris, France) and GSK (Marly le Roi, France). Dr Gwenaëlle Vidal-Trécan and Dr Valérie Buthion participated in a steering committee for a previous study sponsored by GSK (Marly le Roi, France). GSK is purveyor of several oral anticancer drugs.

## Authors’ contributions

Dr LB proposed the original idea of the article and was responsible for the literature review and manuscript writing. PB assisted in the literature review. Dr VB, Dr GV-T and PB assisted Dr LB for discussions of the manuscript content and presentation. All authors have read and approved the final version of the manuscript.

## Pre-publication history

The pre-publication history for this paper can be accessed here:

http://www.biomedcentral.com/1472-6963/14/274/prepub
